# CA199 and CEA expression levels, and minimally invasive postoperative prognosis analysis in esophageal squamous carcinoma patients

**DOI:** 10.1515/med-2024-1127

**Published:** 2025-05-27

**Authors:** Cheng Ji, Lingjia Ji, Fei Wang, Anping Zhang, Liang Shen

**Affiliations:** Department of Thoracic and Cardiac Surgery, Nantong First People’s Hospital, Nantong, Jiangsu, 226000, China; Nursing Group, Affiliated Hospital of Nantong University, Nantong, Jiangsu, 226000, China; Department of Thoracic and Cardiac Surgery, Nantong First People’s Hospital, No. 666 Shengli Road, Chongchuan District, Nantong, Jiangsu, 226000, China

**Keywords:** CA199, CEA, esophageal squamous carcinoma, prognosis analysis

## Abstract

**Background:**

This study explores the factors related to the expression levels of carbohydrate antigen 199 (CA199) and carcinoembryonic antigen (CEA) and their association with poor postoperative prognosis in patients with esophageal squamous cell carcinoma (ESCC) who underwent minimally invasive resection.

**Methods:**

Eighty patients with ESCC who underwent minimally invasive surgery were divided into two groups: 40 with poor prognosis (recurrence) and 40 with good prognosis (no recurrence). Additionally, 80 healthy subjects were selected as a control group. Serum CA199 and CEA levels were measured before surgery and 3 and 6 months postoperatively.

**Results:**

The serum CA199 and CEA levels in the experimental group were significantly higher than in the control group (*P* < 0.05). Patients with poor prognoses within the experimental group had higher CA199 and CEA levels than those with good prognoses (*P* < 0.05). In the poor prognosis group, CA199 and CEA levels at 6 months were significantly higher than at 3 months post-surgery (*P* < 0.05).

**Conclusion:**

Poor prognosis in ESCC patients after minimally invasive resection may be influenced by factors such as lymph node metastasis, lesion length, and tumor location. Elevated CA199 and CEA levels postoperatively can serve as predictors of poor prognosis in patients with ESCC.

## 1 Introduction

With the aging of the population and lifestyle changes, malignant tumors have become one of the leading causes of death in China. According to a survey [1], China’s standardized cancer incidence rate is 201.7/100,000, ranking 68th in the world; at the same time, China’s standardized cancer mortality rate is 130.1/100,000, ranking 12th globally. Esophageal squamous cell carcinoma (ESCC) is one of the most common and fatal malignant tumors in China. It lacks early typical symptoms, is mostly in the middle and late stages when diagnosed, and has a very poor prognosis [2]. Endoscopy, as a major screening technology, can detect ESCC early, but its invasiveness, severe side effects, and dependence on the skills of endoscopists limit its widespread application. People more easily accept simple blood tests and have broad clinical application prospects. Surgery is the main treatment for ESCC. With the development of minimally invasive surgery, thoracoscopic esophagectomy is becoming more and more widely used. For patients who cannot undergo surgery, radiotherapy and chemotherapy are important treatments to control disease progression, which can effectively shrink tumors and reduce the rate of metastasis. Biological and targeted therapy have also shown certain effects in improving the condition. Endoscopic local injection chemotherapy and endoscopic ablation therapy have shown good safety and application prospects in recent years. Treatment can significantly improve the 5-year survival rate for early-stage lesions, and endoscopic minimally invasive resection or ablation therapy is a potential intervention strategy [3,4].

Tumor markers (TM) are closely related to the occurrence and development of malignant tumors and exist in the peripheral blood, other body fluids, or tissues of tumor patients [5]. It can be detected through radioimmunoassay technology, immunofluorescence technology of fluorescently labeled probes, enzyme-linked immunosorbent assay, chemiluminescence immunoassay, chemiluminescence immunoassay, time-resolved fluorescence immunoassay technology, and other methods. TM is divided into tumor embryonic antigens, carbohydrates and glycoproteins, ectopic hormones, enzymes and isozymes, tumor antigens, oncogenes, tumor suppressor gene protein products, etc. Its expression level can help diagnose malignant tumors, monitor recurrence, or evaluate disease efficacy and prognosis [6]. An ideal TM should have high sensitivity and specificity and be able to localize and differentially diagnose tumors. No ESCC-specific TM has been found yet, and biomarkers such as carcinoembryonic antigen (CEA), squamous cell carcinoma antigen (SCC), and Cyfra21-1 are common methods for clinical auxiliary diagnosis of ESCC [7].

Carbohydrate antigen 19-9 (CA 19-9). CA199 is a mucin antigen with a relative molecular mass of 10,000 in the form of monosialoganglioside [8]. The serum levels of CA199 are elevated in gastrointestinal tumors such as esophageal cancer, gastric cancer, colorectum, etc., but there is a lack of specific evidence to diagnose esophageal cancer [9].

CEA was first discovered in 1965 and is one of the earliest discovered and most studied TMs [10]. Some studies have shown that the increase in serum CEA has certain significance for the diagnosis and prognosis of ESCC and can be used as an auxiliary clinical diagnostic indicator for ESCC [11].

The single diagnostic value of TMs is low, and combined diagnosis can improve sensitivity and specificity. Studies have shown that when single CEA, SCC, Cyfra21-1, and NSE tests were performed on patients with esophageal cancer, the positive rates were 36.0, 53.5, 26.7, and 24.4%, respectively. When combined with detection, its sensitivity and specificity can reach 82.6 and 83.7% [12]. It is reported that the positive rates of Cyfra21-1, CA199, and SCC for esophageal cancer are 58.1, 40.5, and 31.1%, respectively [13]. It can be seen that the joint detection of multiple TMs can significantly improve diagnostic efficiency.

Minimally invasive resection is an important way to treat ESCC. It is suitable for treating esophageal cancer of different stages and locations and is of great significance in prolonging the survival of patients [14,15]. However, some studies have pointed out that due to the large tumor lesions and wide range of tumor invasion, ESCC patients still have a high risk of recurrence after minimally invasive resection, which seriously affects the prognosis [16,17]. Therefore, it is important to explore the factors that may influence the poor postoperative prognosis of ESCC patients who undergo minimally invasive resection. Studies have shown that the poor postoperative prognosis of patients with stage III thoracic esophageal cancer may be related to the patient’s age, gender, and TNM stage [18,19,20].

However, the influencing factors obtained from the above studies cannot guide the formulation of intervention measures for patients with poor prognosis of ESCC. This study explores the expression levels of CA199 and CEA in ESCC patients who underwent minimally invasive resection and explores the factors associated with the impact of these two TMs on poor postoperative prognosis. It aims to lay a clinical data foundation for reducing the occurrence of adverse prognostic reactions in future ESCC patients.

## 2 Information and methods

### 2.1 Normal information

Eighty patients collected from June 2022 to January 2024 underwent minimally invasive cutting in our hospital and completed 1 year of follow-up after surgery as the experimental group. The clinical data of 40 patients in the experimental group were selected in the experimental group, as a poor prognosis group. The clinical data of esophageal squamous carcinoma patients who received minimally invasive resection during the hospital received minimally invasive resection during the same period and completed a one-year follow-up (uncompromising) is used as a good prognosis group. At the same time, 80 health tests were selected as the control group. This study has passed the ethical approval of our court.

### 2.2 Inclusion criteria

Diagnostic standards meet the “Guidelines for Standardizing the Diagnosis and Treatment of esophageal cancer” [21]. The diagnostic criteria of esophageal squamous carcinoma are related to the diagnosis of esophageal squamous carcinoma.

(1) Anophayal surgical pathological examination is diagnosed as esophageal squamous carcinoma
(2) first time of surgical treatment
(3) Complete information.

### 2.3 Exclusion criteria

1. Those who have mental illness or intellectual developmental disorders.
2. Severe liver and kidney insufficiency or heart failure and severe organ dysfunction.
3. Combined serious coronary heart disease and diabetes.
4. Autoimmune or connective tissue disease.
5. Acute phase of cerebral infarction.
6. Malignant tumors
7. Those who have received surgery, radiotherapy, or chemotherapy.

## 3 Method

### 3.1 Prognostic assessment methods

The examination results are recorded at 3, 6, 9, and 12 months of follow-up. CA199 and CEA levels were measured at 3 and 6 months after surgery. If recurrence is indicated by CT or pathological examination, it is classified as a poor prognosis and is included in the poor group; otherwise, it is included in the good group.

### 3.2 Baseline data: Designed and compiled by the researcher

① General information: Gender (male, female), age, TNM stage. [Stage I, stage II, and stage III refer to the tumor TNM stage revised by the American Joint Committee on Cancer (AJCC) [7]. Stage I: the lesion length is <3 cm and invades the mucosa and submucosa and has no local lymph node metastasis. Stage II: The lesion is 3–5 cm long and invades part of the muscle layer without lymph node metastasis. Stage III: The length of the lesion is >5 cm, and it invades the whole muscle layer or has external invasion, and there is local lymph node metastasis.] The degree of differentiation (poor differentiation, moderate differentiation, and high differentiation refers to the World Health Organization (WHO) [8] esophageal cancer histological differentiation standards). Lymph node metastasis (present or absent), lesion length, and tumor location (upper, middle, and lower segment).

② Laboratory test data: 3 mL of fasting venous blood was collected from the patient before surgery, 3 months, and 6 months after surgery, and centrifuged (speed: 3,000 rpm, centrifugal radius: 15 cm, centrifugation time: 10 min). The upper serum was separated, and enzyme-linked immunoassay was used to determine carbohydrate antigen 199 (CA199) levels and CEA. The kits were purchased from Shanghai Enzyme Biotechnology Co., Ltd. and operated according to the instructions.

### 3.3 Statistical analysis

The data in this study were analyzed and processed by SPSS21.0 statistical software. The measurement data were expressed as (average ± SD), the *t*-test was used, and the count data were expressed as the % rate. The *χ*2 test was adopted. The logistic regression model was used to analyze the impact of CEA and CA199 on the prognosis of patients with ESCC. The receiver operating characteristic (ROC) curve was used to analyze the effectiveness of CEA and CA199 in predicting poor prognosis in patients with ESCC. *P* < 0.05 indicates that the difference is statistically significant.

We used logistic regression analysis to observe the correlation of adverse prognostic factors. The postoperative prognosis of patients with ESCC who underwent minimally invasive resection was taken as the dependent variable, where poor outcomes were assessed as ‘0’ and good outcomes as ‘1’. The independent variables included all clinical variables, as shown in Table 1, and CEA and CA199 levels from Table 3 were individually analyzed through single-factor logistic regression. *P*-values were relaxed to <0.2, and qualified variables (lesion length, lymph node metastasis, tumor location, CA199, and CEA) were included as independent variables to establish a multiple regression model.

**Table 1:** Comparison of general clinical data of patients

Index	Experimental group (*n* = 80)		Control group (*n* = 80)	*t*/*x* ^2^	*P*
	Poor prognosis group (*n* = 40)	Good prognosis group (*n* = 40)			
Average age	56.95 ± 8.72	56.43 ± 8.67	56.40 ± 6.74	0.267	0.790
Gender (male/female)	19/21	23/17	45/35	0.802	0.370
Lesion length (cm)	5.03 ± 1.02	3.25 ± 0.83	—	8.561	<0.001
TNM stage [*n* (%)]				0.840	0.657
Phase II	8 (20.00)	6 (15.00)	—		
Phase Ⅲ	18 (45.00)	22 (55.00)	—		
Phase Ⅳ	14 (35.00)	12 (30.00)	—		
Differentiation [*n* (%)]				0.216	0.898
Poorly differentiated	8 (20.00)	9 (22.50)	—		
Moderately differentiated	15 (37.50)	16 (40.00)	—		
Highly differentiated	17 (42.50)	15 (37.50)	—		
Tumor location				11.823	0.003
Upper section	16 (40.00)	3 (7.50)	—		
Middle section	15 (37.50)	25 (62.50)	—		
Lower section	9 (22.50)	12 (30.00)	—		
Lymph node metastasis				6.050	0.014
Yes	13 (32.50)	4 (10.00)	—		
No	27 (67.50)	36 (90.00)	—		


**Ethical approval:** This study was approved by the ethical committee of Nantong First People’s Hospital. Informed consent was obtained from all participants.

## 4 Results

### 4.1 Comparison of clinical data

We compared the groups’ general clinical data of selected patients (Table 1). Compared to the lesion length, lymph node metastasis, and tumor location between the poor prognosis group and the good prognosis group, the differences were statistically significant (*P* < 0.05), and the TNM stage and degree of differentiation were comparable (*P* > 0.05). Moreover, the gender, age, and other information between groups are comparable (*P* > 0.05).

### 4.2 Comparison of CEA and CA199 in the experimental group and control group

**Figure 1:**
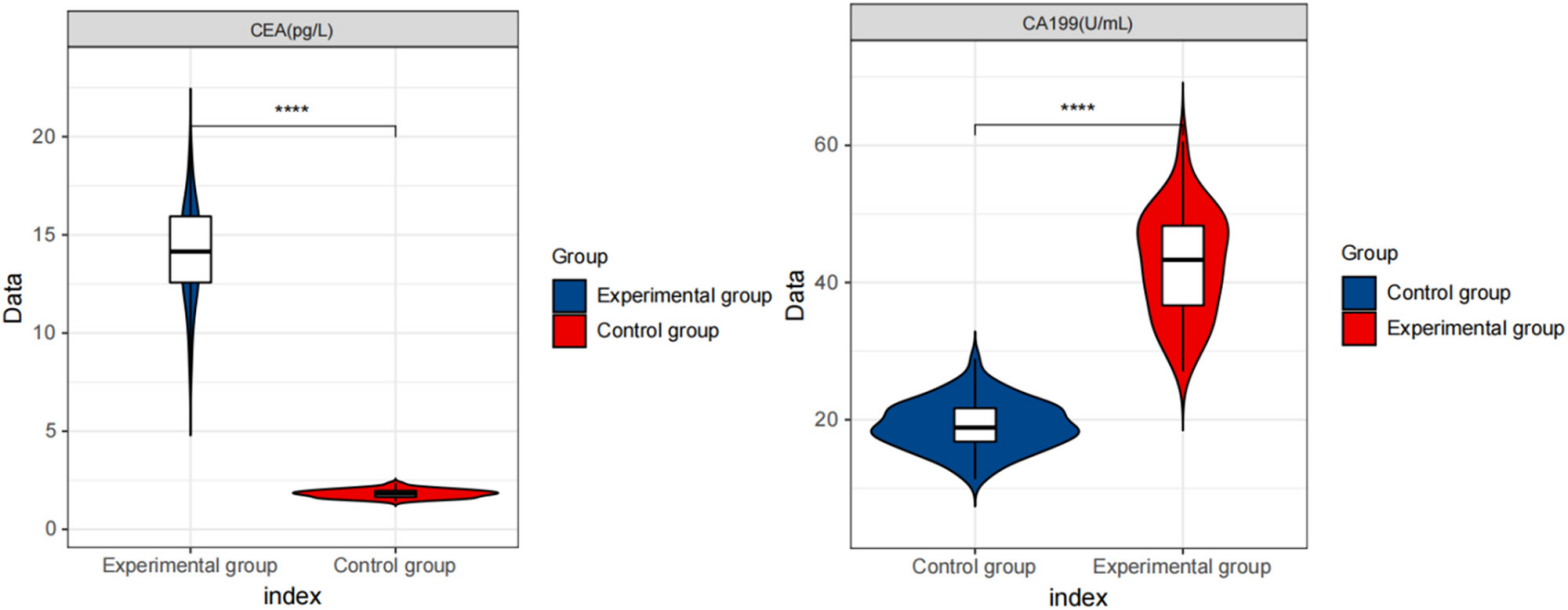
Violin plot for comparison of CEA and CA199 levels among two groups. This figure illustrates the distribution and differences in CEA and CA199 levels between the two groups using a violin plot, highlighting each group’s central tendency and variability.

We measured CEA and CA199 levels between the two groups. The results showed (Table 2 and Figure 1) that the serum CEA and CA199 levels of the experimental group were higher than those of the control group (*P* < 0.05).

**Table 2:** Comparison of the two indices among the two groups (*x* ± *s*)

Index	Experimental group (*n* = 80)	Control group (*n* = 80)	*t*/*x* ^2^	*P*
CEA (pg/L)	14.04 ± 2.51	1.82 ± 0.21	43.394	<0.001
CA199 (U/mL)	42.66 ± 7.66	19.15 ± 3.52	24.944	<0.001

Note: CA199: carbohydrate antigen 199; CEA, carcinoembryonic antigen.

### 4.3 Comparison of CEA and CA199 in the poor and good prognosis groups

**Figure 2:**
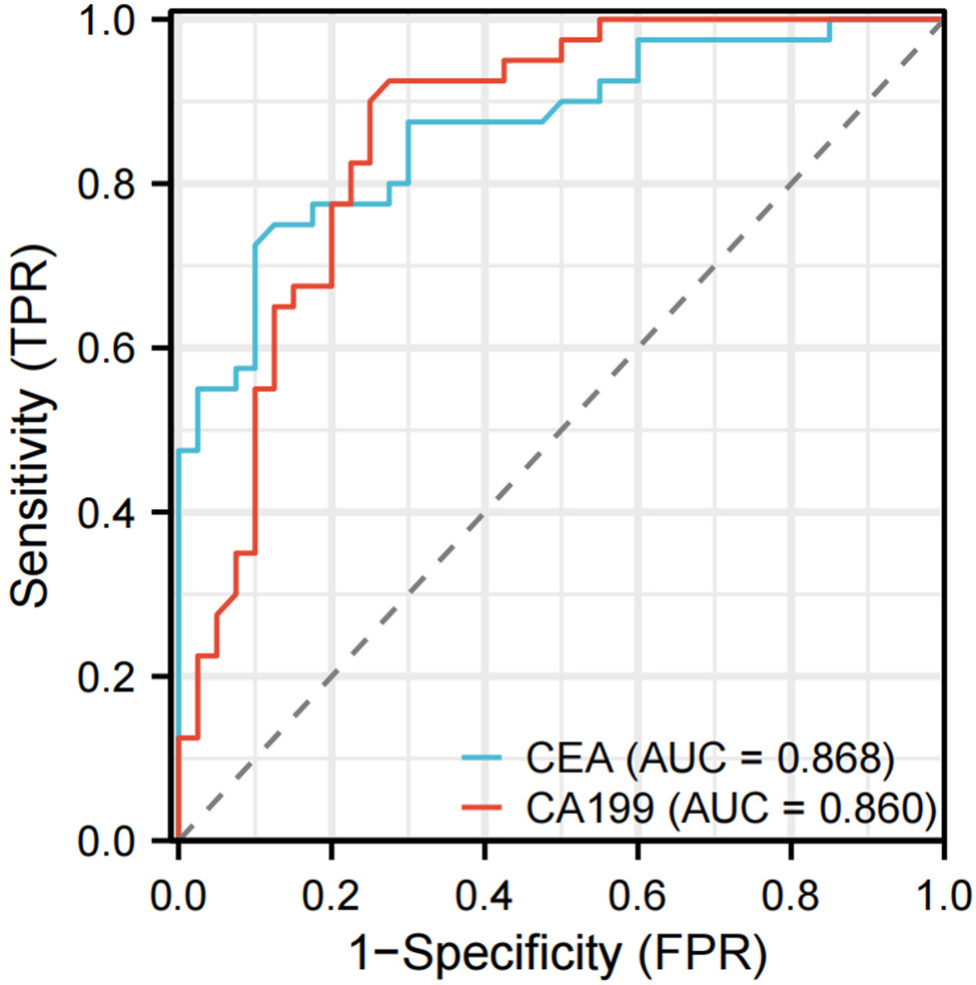
ROC curve analysis. It demonstrates the diagnostic performance of the selected biomarkers, showing sensitivity versus 1-specificity across different thresholds.

We also compared the expressions of CEA and CA199 in the experimental group’s poor prognosis and good prognosis. The results showed (Table 3 and Figure 2) that 3 months after surgery, the CEA and CA199 levels in the poor prognosis group and the good prognosis group were comparable (*P* > 0.05). Six months after surgery, the serum CEA and CA199 levels of the poor prognosis group were higher than those of the good prognosis group (*P* < 0.05). In the poor prognosis group, CEA and CA199 levels 6 months after surgery were higher than those 3 months after surgery (*P* < 0.05).

**Table 3:** Comparison of the levels of CEA and CA199 in the poor and good prognosis group (*x* ± *s*)

Index	Time	Poor prognosis group (*n* = 40)	Good prognosis group (*n* = 40)	*t*/*x* ^2^	*P*
CEA (pg/L)	Before surgery	14.08 ± 2.58	14.03 ± 2.49	0.088	0.930
	3 months after surgery	3.05 ± 0.65*	3.01 ± 0.61*	0.284	0.777
	6 months after surgery	5.96 ± 1.14*	3.85 ± 1.52*	7.024	0.000
CA199 (U/mL)	Before surgery	42.71 ± 7.71	42.64 ± 7.67	0.041	0.968
	3 months after surgery	23.05 ± 4.25*	23.07 ± 4.18*	-0.021	0.983
	6 months after surgery	31.52 ± 5.74*	24.11 ± 4.23*	6.573	0.000

*Note: Means comparison within-group before surgery, *P* < 0.05.

### 4.4 Logistics regression analysis of related factors with poor prognosis effects

The results showed that lymph node metastasis, long lesions, upper segment tumors, CA199, and CEA overexpression were all factors affecting the poor prognosis of patients with ESCC after minimally invasive resection (*P* < 0.05) (Table 4).

**Table 4:** Results of univariate and multivariate logistic regression analysis

Index	OR (95% CI) univariate analysis	*P* value univariate analysis	OR (95% CI) multivariate analysis	*P* value multivariate analysis
Age	0.993 (0.943–1.045)	0.785		
Lesion length	0.127 (0.053–0.304)	<0.001	0.025 (0.001–0.403)	0.009
stage	1.294 (0.467–3.590)	0.620		
Differentiation	1.630 (0.477–5.565)	0.436		
Location	8.889 (2.215–35.665)	0.002	42.002 (3.803–198.118)	0.044
Lymph node metastasis	0.231 (0.068–0.787)	0.019	0.113 (0.001–0.457)	0.035
CEA	0.303 (0.180–0.508)	<0.001	0.225 (0.064–0.792)	0.020
CA199	0.727 (0.632–0.837)	<0.001	0.708 (0.524–0.955)	0.024

### 4.5 Predictive performance analysis

ROC curve analysis showed that the AUC)of serum CEA and CA199 in predicting poor prognosis in patients with ESCC were 0.868 and 0.860, respectively. It is suggested that these two indicators have high predictive value in the prognosis assessment of ESCC.

## 5 Discussion

At this stage, surgery is the main method for treating esophageal cancer. According to relevant research reports, esophageal cancer still has a high recurrence rate after surgical treatment, among which the recurrence rate of esophageal adenocarcinoma is as high as 50.00%. In comparison, the recurrence rate of ESCC is 34.00–79.00% [22].

Clinically, minimally invasive resection can improve the therapeutic effect of ESCC. However, some studies have pointed out that some patients have a higher recurrence rate after minimally invasive resection, which increases the risk of poor prognosis [23]. Some researchers [10] conducted a follow-up analysis on patients with thoracic ESCC who underwent radical resection, and the results showed that the postoperative recurrence rate was 34.6%[24]. It is suggested that ESCC patients have a higher risk of poor prognosis after surgery.

Therefore, it is necessary to find factors related to poor prognosis after minimally invasive resection of ESCC. Under these conditions, finding sensitive indicators to monitor patient’s condition changes after ESCC surgery is the key to improving prognosis and survival rates. CEA is a tumor embryonic antigen with acidic glycoprotein as the main component [25], and CA199 is a mucin antigen with a relative molecular mass of 10,000 in the form of monosialoganglioside [26].

The results of this study show that lymph node metastasis, long lesions, and upper segment tumors are all factors affecting the poor prognosis of ESCC patients after minimally invasive resection.

The reasons are analyzed as follows. ① Lymph node metastasis: lymph from each esophageal segment of the body will normally be injected into nearby lymph nodes, making lymph node dissection more difficult during minimally invasive resection. Especially when metastasis occurs in the thoracic lymph nodes, it is very likely to move up to the upper mediastinal lymph nodes and metastasize to the abdominal lymph nodes. However, effective dissection cannot be performed during minimally invasive surgery, resulting in some tumor cells remaining in the body, increasing the risk of poor prognosis [27,28]. In this regard, it is recommended that patients with preoperative lymph node metastasis should focus on lymph node dissection during minimally invasive resection. At the same time, corresponding adjuvant treatment can be taken after surgery to improve further the clinical treatment effect, which may have a positive significance in reducing the risk of poor prognosis in ESCC patients after minimally invasive resection.

② Longer lesion length: On the one hand, the long diameter of the tumor can affect the surgical resection rate; on the other hand, the size of the tumor is related to the degree of infiltration and lymph node metastasis. As the diameter of the tumor increases, the risk of lymph node metastasis increases, and the degree of tumor infiltration will continue to increase, affecting the patient’s prognosis [29]. In this regard, it is recommended that ESCC patients with long lesions increase the surgical resection length during minimally invasive resection to clear the lesions as much as possible to improve the prognosis.

③ Upper segment tumors: Most ESCC in the upper thoracic segment are in the T4 stage, and the tumors often invade the structures around the esophagus. Moreover, the operation is more difficult and riskier since the upper chest segment is connected to the trachea and recurrent laryngeal nerve. The surgical margins are prone to residual cancer tissue, which increases the risk of poor patient prognosis [30,31]. In this regard, it is recommended that the scope of surgical resection and lymph node dissection should be determined based on the actual tumor location of ESCC patients to improve the effect of surgical treatment and improve the prognosis of patients.

④ Serum CA199 and CEA are overexpressed: CA199 and CEA are markers related to esophageal cancer. If the preoperative serum CA199 and CEA expression in ESCC patients is too high, it may indicate that the tumor is severely malignant and has poor biological behavior. It mostly manifests as infiltrative growth and easily invades the serosa, which directly affects the effect of minimally invasive surgical treatment and increases the risk of poor prognosis for patients [31,32,33]. In this regard, it is recommended that for patients with over-expressed serum CA199 and CEA before surgery, adjuvant treatment can be performed before surgery to reduce the levels of CA199 and CEA. Waiting until the ideal state is reached before performing surgery may have positive significance in reducing the poor prognosis of ESCC patients after minimally invasive resection.

In this study, CA199 and CEA levels 6 months after surgery were also used for correlation analysis. The results also suggest that the postoperative increases in CA199 and CEA levels can be used as predictors of poor prognosis in patients with ESCC after minimally invasive resection. ROC curve analysis showed that the AUC of serum CEA and CA199 in predicting poor prognosis in patients with ESCC were 0.868 and 0.860, respectively. It is suggested that these two indicators have high predictive value in the prognosis assessment of ESCC.

This study also has some limitations. The sample size of this study was too small, and it was a single-center study. The sample size can be expanded, and multicenter studies can be conducted. This study only focused on two types of TM, and more TMs can be combined for future research. To explore the relationship between TM and ESCC in more depth and to provide clinical help for the prognosis of ESCC patients.

In summary, lymph node metastasis, lesion length, tumor location, and serum CA199 and CEA levels may be factors affecting the poor prognosis of ESCC patients after minimally invasive resection. In this regard, clinical practice should select the most appropriate treatment measures based on the patient’s situation to improve the patient’s prognosis.
